# Urine levels of HMGB1 in Systemic Lupus Erythematosus patients with and without renal manifestations

**DOI:** 10.1186/ar4015

**Published:** 2012-08-14

**Authors:** Deena A Abdulahad, Johanna Westra, Johannes Bijzet, Sebastian Dolff, Marcory C van Dijk, Pieter C Limburg, Cees GM Kallenberg, Marc Bijl

**Affiliations:** 1 Department of Rheumatology and Clinical Immunology, University Medical Center Groningen, University of Groningen, PO Box 30.001, 9700RB Groningen, The Netherlands; 2 Pathology and Laboratory Medicine, University Medical Center Groningen, University of Groningen, PO Box 30.001, 9700RB Groningen, The Netherlands; 3 Department of Pathology, University Medical Center Groningen, University of Groningen, PO Box 30.001, 9700RB Groningen, The Netherlands; 4 Department of Internal Medicine and Rheumatology, Martini Hospital, PO Box 30.033, 9700RM Groningen, The Netherlands; 5 Department of Nephrology, University Hospital Essen, University Duisburg-Essen, 47057 Duisburg, Germany

## Abstract

**Introduction:**

Lupus nephritis (LN) is a severe and frequent manifestation of systemic lupus erythematosus (SLE). Its pathogenesis has not been fully elucidated but immune complexes are considered to contribute to the inflammatory pathology in LN. High Mobility Group Box 1 (HMGB1) is a nuclear non-histone protein which is secreted from different types of cells during activation and/or cell death and may act as a pro-inflammatory mediator, alone or as part of DNA-containing immune complexes in SLE. Urinary excretion of HMGB1 might reflect renal inflammatory injury. To assess whether urinary HMGB1 reflects renal inflammation we determined serum levels of HMGB1 simultaneously with its urinary levels in SLE patients with and without LN in comparison to healthy controls (HC). We also analyzed urinary HMGB1 levels in relation with clinical and serological disease activity.

**Methods:**

The study population consisted of 69 SLE patients and 17 HC. Twenty-one patients had biopsy proven active LN, 15 patients had a history of LN without current activity, and 33 patients had non-renal SLE. Serum and urine levels of HMGB1 were both measured by western blotting. Clinical and serological parameters were assessed according to routine procedures. In 17 patients with active LN a parallel analysis was performed on the expression of HMGB1 in renal biopsies.

**Results:**

Serum and urinary levels of HMGB1 were significantly increased in patients with active LN compared to patients without active LN and HC. Similarly, renal tissue of active LN patients showed strong expression of HMGB1 at cytoplasmic and extracellular sites suggesting active release of HMGB1. Serum and urinary levels in patients without active LN were also significantly higher compared to HC. Urinary HMGB1 levels correlated with SLEDAI, and showed a negative correlation with complement C3 and C4.

**Conclusion:**

Levels of HMGB1 in urine of SLE patients, in particular in those with active LN, are increased and correlate with SLEDAI scores. Renal tissue of LN patients shows increased release of nuclear HMGB1 compared to control renal tissue. HMGB1, although at lower levels, is, however, also present in the urine of patients without active LN. These data suggest that urinary HMGB1 might reflect both local renal inflammation as well as systemic inflammation.

## Introduction

Systemic lupus erythematosus (SLE) is a prototypic systemic autoimmune disease characterized by a wide array of autoantibodies, mainly against nuclear components. Autoantibody production is associated with various clinical manifestations and among these manifestations, renal involvement, that is, lupus nephritis (LN), is the most serious clinical problem predicting morbidity and mortality [1,2]. The mechanisms underlying the pathogenesis of LN are not fully elucidated. However, LN has often been considered an inflammatory disease resulting from deposition of preformed immune complexes or binding of autoantibodies to antigens localized to glomeruli, so-called *in situ *complex formation [3-5]. Among the many antibodies potentially participating in the formation of immune complexes, antibodies against DNA are the hallmark of SLE. Recently, it has been shown that these DNA-containing immune complexes constitute among others, high mobility group box 1 (HMGB1), which has been suggested to be involved in binding of these immune complexes to renal tissue and initiate renal injury [6].

HMGB1 is a nuclear DNA-binding protein that resides inside the nucleus and can be released to the extracellular space under specific conditions [7,8]. Whereas HMGB1 is actively released from lipopolysaccharide (LPS), TNF-, and IL-1 activated monocytes and macrophages, its release also occurs passively during the late phase of apoptosis as well as during necrosis [7,9,10]. Extracellularly, HMGB1 acts as an alarmin involved in inflammatory reactions through binding to its functional receptors, that is the receptor of advanced glycation end products (RAGE) and toll-like receptors (TLR)-2, -4, and -9 [11-14].

There is accumulating evidence that HMGB1 contributes to the pathogenesis of inflammatory and autoimmune diseases, especially SLE [15-17]. This is related to the fact that apoptotic cells accumulate in SLE and are the main source of autoantigens, including HMGB1 [7,18]. We and others showed that serum levels of HMGB1 are elevated in SLE patients and correlate with SLE disease activity score and, inversely, with levels of the complement components C3 and C4. Moreover, we could demonstrate that serum levels of HMGB1, in particular, were increased in SLE patients with active renal disease and correlated with proteinuria [15]. The origin of the increased serum levels of HMGB1 is not known, and HMGB1 could possibly result from release from damaged and/or inflamed renal tissue. As such, HMGB1 could appear in the urine during (active) LN. In this study, we hypothesize that urinary excretion of HMGB1 reflects renal inflammatory injury in SLE. We investigated this by measuring HMGB1 levels in the urine of SLE patients and correlating this to clinical and biochemical measures of renal and systemic disease activity.

## Materials and methods

### Patients

Sixty-nine patients (nine male, sixty female), median age 41 (range 16 to 81) years, who fulfilled at least four of the American College of Rheumatology (ACR) revised criteria for the diagnosis of SLE, and seventeen healthy controls (HC) (two male, fifteen female), median age 26 (range 20 to 59) years were enrolled in the study. The study was approved by the Medical Ethical Committee of the University Medical Center Groningen, University of Groningen, the Netherlands, and all patients and HC gave informed consent. Clinical data were obtained from all patients and the study was conducted according to the ethical guidelines of our institution in accord with the Declaration of Helsinki. Among the patients, 21 had biopsy-proven active LN, 15 had a history of LN without current activity, and 33 had non-renal SLE (Table 1). Disease activity at the time of blood sampling was assessed by the SLE disease activity index (SLEDAI). Currently active LN (n = 21) was defined as proliferative glomerulonephritis (class III or IV) in a parallel-obtained renal biopsy (n = 17) or as the presence of an active urinary sediment representing glomerular injury in a patient with biopsy-proven LN before. Blood and urine was obtained when patients were admitted, at a maximum of 3 days before a renal biopsy was taken. Further characteristics of the patients are summarized in Table 1.

**Table 1:** Demographic, clinical and laboratory data of systemic lupus erythematosus (SLE) patients at the time of the study

	Active LN	No active LN	
		History of LN	No history of LN
**Total number**	21	15	33
**Male/female, number**	4/17	3/12	2/31
**Age, years, median (range)**	38 (16 to 53)	46 (18 to 61)	48 (20 to 81)
**SLEDAI, mean ± SD**	13.7 ± 3.7 ***^,+++^	2.7 ± 1.7	2.8 ± 2.7
**Anti-dsDNA antibody, IU/ml, median (range)**	140 (3 to 1,000) **^, +^	19 (3 to 432)	20.5 (3 to 1000)
**C3, g/l, median (range)**	0.55 (0.27 to 1.13)***^,++^	0.93 (0.43 to 1.48)	0.97 (0.31 to 11.4)
**C4 (g/l), median (range)**	0.08 (0.03- 0.3)*^,+^	0.16 (0.06 to 0.3)	0.12 (0.02 to 1.04)
**CRP, mg/I, median (range)**	5 (5 to 121)	5 ( 5 to 21)	5 (5 to 33)
**eGFR, ml/min**	72 (24 to 142)	81 (45 to 145)	89 (31 to 140)
**Serum creatinine, µmol/l, median (range)**	75 (46 to 406)	71 (51 to 126)	62 (47 to 141)
**24-hr proteinuria, g/24hr**	1.5 (0.3 to 8.4)***^,+++^	0.3 (0.2 to 2.5) *^# # #^*	0.0 (0.0 to 0.1)
**Urine creatinine, mmol/l, median (range)**	7.6 (0.6 to 21.3)	4.3 (1.6 to 15.7)	6.2 (0.7 to 14.9)
**With/without treatment, number**	12/9	13/2	21/12
**Patients using prednisone, number (%), dose, mg/day, median (range)**	10 (47.6%) 7.5 (2.5 to 60)	10 (66.6%) 10 (5 to 60)	8 (24.2%) 5 (1.25 to 10)
**Patients taking hydroxychloroquine, number (%), dose, mg/day, median (range)**	8 (38%) 350 ( 200 to 600)	2 (13.3%) 400 (400)	14 (42.2%) 400 (200 to 600)
**Patients using azathioprine, number (%), dose, mg/day, median (range)**	3 (14.3%) 100 (75 to 150)	7 (46.6%) 75 (50 to150)	6 (18.2%) 125 (75 to 150)

^+^Indicates difference between active lupus nephritis (LN) patients and patients with no active LN but with history of LN (^+^P ≤ 0.05, ^++^*P *≤ 0.005, ^+++^*P *≤ 0.0005). *Indicates difference between active and inactive SLE patients (*P = 0.05,**P ≤ 0.005, ***P ≤ 0.0005). ^#^Indicates difference between patients without active LN but with history of LN and inactive SLE patients with no history of LN (^#^*P *= 0.05, ^##^*P *≤ 0.005, ^###^*P *≤ 0.0005). Anti-dsDNA: anti-double stranded DNA; C3: Complement 3; C4 Complement 4; CRP: C-reactive protein; eGFR: estimated glomerular filtration rate; SLEDAI: systemic lupus erythematosus disease activity index.

### Materials

Serum and urine samples were collected from patients and HC. Patients' urine samples that were nitrite-positive urine samples on a dip stick test or demonstrated evidence of bacterial contamination in the sediment were excluded. Levels of anti-dsDNA, C-reactive protein (CRP), creatinine (Cr), and complement factors (C3, C4) in sera, and 24 hr proteinuria and urine Cr levels were determined by routine techniques.

Paraffin-embedded sections of renal biopsy specimens obtained from 17 patients with active LN were included in the present study. Renal tissues from three unaffected parts of the kidneys of patients with renal cell carcinoma were used as controls.

### Western blot for serum and urine HMGB1

Serum and urine samples from SLE patients and HC were collected and analyzed for HMGB1 by Western Blotting as described previously. Western blot was chosen because commercially available ELISA kits are not suitable for SLE sera, due to interference of autoantibodies (15). Also these kits are not recommended for urine samples. Reproducibility was calculated over the positive control in eight blots performed on different days and coefficient of variation was 22.1%.

Urine samples were concentrated between 30 and 300 times using Vivaspin 15R (Sartorius Stedim Biotech, Gottingen, Germany). Serum and urine samples from SLE patients and HC were diluted in SDS buffer (0.063 M Tris.HCl pH 6.8, 2% SDS, 10% glycerol, 0.015% BromePhenol Blue, and 5% ß-mercaptoethanol) and heated at 98ºC for five minutes. The volume of urine loaded to the gel was corrected for the concentration factor. Next, proteins were resolved on 12.5% SDS-PAGE gel (Criterion gel BioRad, Veenendaal, The Netherlands) and transferred to polyvinylidene fluoride membrane (Millipore, Amsterdam, The Netherlands) followed by incubation with anti-HMGB1 mouse monoclonal antibody (1:250; R&D Systems, Abingdon, UK). Detection was done with polyclonal goat anti-mouse IgG labelled with IRDye800 (1: 5000; LI-COR Biotechnology, Westburg, Leusden, the Netherlands). Blots were scanned with Odyssey infrared Imaging System (LI-COR Biotechnology) and then analyzed with the Odyssey software (version 2.1). A standard sample was obtained from lyzed human keratinocyte HaCaT cells and included in each blot as an internal control. In each blot, levels of HMGB1 were expressed as values of fluorescence intensity and were normalized against the standard sample. Urinary HMGB1 was expressed as HMGB1/Cr ratio (intensity units/mmol/l) to correct for differences in dilution.

### Analysis of renal biopsies

Biopsies concomitantly taken with serum and urine samples were processed. All biopsies were reviewed and classified by an experienced nephropathologist (MCvD) according to the revised criteria for LN [19]. The activity index (AI) and chronicity index (CI) were calculated for each specimen with maximum scores of 24 for the AI and 12 for the CI [19]. Histological data are shown in Table 2.

**Table 2:** Histological data of renal biopsies from seventeen systemic lupus erythematosus (SLE) patients with active renal disease and three controls

Number	Sex	ISN class	Activity index(AI)	Chronicity index(CI)	HMGB1-negative nuclei in renal tissues, %
1	F	IV	10	3	35.0
2	F	III	3	7	40.0
3	F	III	2	0	25.0
4	F	IV	8	0	20.4
5	F	III	3	0	28.1
6	F	III	1	4	29.4
7	F	IV	5	0	37.2
8	F	IV	3	2	24
9	F	IV	5	3	38
10	F	III	4	3	26
11	F	IV	3	0	42
12	F	III	2	0	0.7
13	F	III	7	2	31.1
14	M	III	3	0	12
15	M	na	1	0	19.1
16	F	IV	6	2	26.3
17	F	IV	8	3	26.3
18 Control	F	-	-	-	0
19 Control	F	-	-	-	0
20 Control	F	-	-	-	0

M: male; F: female; ISN: International Society of Nephrology; HMGB1: high mobility group box 1; na: not assessed.

### Immunohistochemical staining of renal biopsies

Kidney sections (4 µm) were used for all staining experiments. Sections were deparaffinised. Next, antigen retrieval and endogenous peroxidase blocking was performed. Slides were incubated with rabbit anti-HMGB1 antibody (Abcam, Cambridge, UK), goat anti-RAGE antibody (AbD Serotec, Dusseldorf, Germany), rabbit anti-TLR2 antibody (Abcam), and rabbit anti-TLR-4 antibody (Abcam). Subsequently, slides were incubated with HRP-labelled secondary antibodies (DakoCytomation, Glostrup, Denmark). Next, slides were incubated in diaminobenzidine solution and counterstained with hematoxylin. Using ImageScope software (Aperio Technologies, Vista, CA, USA) morphometry was performed on entire renal sections stained with antibodies against RAGE, TLR2 and TLR4.

### Analysis of immunohistochemical staining

#### Evaluation of HMGB1 staining

The stained slides were coded and analyzed independently by two observers (DA and JW) in a blinded fashion. Cellular distribution of HMGB1 was determined in the kidney by counting one hundred nuclei in three brightfield pictures and scoring both HMGB1-positive (brown) and HMGB1-negative (blue) nuclei. Results are expressed as the percentage of negative cells.

#### Evaluation of TLR2, TLR4 and RAGE staining

Expression of TLR 2, TLR4 and RAGE was analyzed on entire renal tissue sections of active LN patients and controls stained with antibodies against TLR2, TLR4 and RAGE.

Glass slides were digitally scanned with 20× objective using Aperio ScanScope (Aperio Technologies). The images were quantitatively analyzed and enhanced using the computer software system ImageScope (Aperio Technologies) for histological examination. The positive pixel count algorithm was used to quantify the amount of a specific stain present in a scanned image. Colour (range of hues and saturation) and intensity were specified. For pixels that satisfied the colour specification, the algorithm counted the number and intensity sum in each intensity range. Positive colour class was divided into three intensity ranges producing four categories of staining, namely strong, medium, weakly positive, and negative staining. Each pixel that stained was put into one of the four categories and total pixel counts were reported, along with intensity, for each category. The percentage of positivity was calculated by using the average of positive intensities divided by the total numbers of positively and negatively stained pixels.

### Statistical analysis

Data are presented as median (range) unless stated otherwise. Statistical analysis was performed by using the statistical package Graph Pad Prism, version 3.02 (Graph Pad software Inc., San Diego, CA, USA). A student Mann-Whitney test was performed for comparison of different groups as appropriate. Spearman rank correlation was used to assess correlations. A *P- *value < 0.05 was considered significant.

## Results

### Serum and urine HMGB1 levels

**Figure 1:**
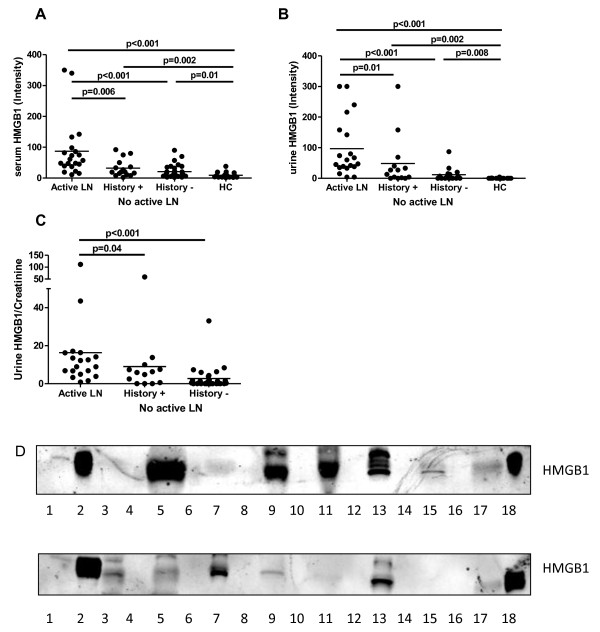
**High mobility group box 1 (HMGB1) concentrations in serum and urine from SLE patients with active lupus nephritis (LN), patients without active LN but with a history of LN, patients without history of LN, and healthy controls (HC)**. (**A**) Serum HMGB1 levels. (**B**) Urine HMGB1 levels. (**C**) Urine HMGB1 levels corrected for urine creatinine levels. Horizontal lines represent the median. (**D**) Representative blots for urine HMGB1 measurement; lane 1: molecular weight marker, lanes 2, and 18: positive control, lanes 3, 5, 7, 9, 11, 13, and 15: urine samples of SLE patients, lane 17: urine of HC.

Serum HMGB1 levels in active LN patients were significantly increased (intensity 57, range 11 to 350) compared to HC (intensity 6, 1 to 38) and also compared to patients without active LN (intensity 16, 2 to 92) (Figure 1A). Patients without active LN, but with a history of renal involvement had relatively higher levels of serum HMGB1 (intensity 20, 2 to 80) compared to those without renal involvement (intensity 13, 2 to 92), but this difference was not statistically significant.

Representative blots of urine measurements of HMGB1 are shown in Figure 1D. In lanes 3, 5, 7, 9, 11, 13 and 15, patients' samples were run (even lanes were left empty), while in lanes 2 and 18 the positive control is shown. In each blot a urine sample of a HC was run in lane 17, whereas a molecular weight marker was run in lane 1. Urinary HMGB1 levels were significantly increased in patients with active LN (intensity 54, 4 to 300) compared to HC (intensity 0, 0 to 4) and to patients with no active LN (intensity 4, 0 to 300) (Figure 1B). In patients with no active LN but having a history of LN, levels of urinary HMGB1 were increased (intensity 20, 0 to 300), but not significantly compared to patients without a history of LN (1, 0 to 87) (Figure 1B). To correct for dilution, urinary levels were expressed as HMGB1/Cr ratios. In Figure 1C these ratios are shown in the patient groups, and also here a significant difference was seen between patients with active LN and patients without active LN.

### Correlation of urine HMGB1 levels with clinical and serological findings

**Figure 2:**
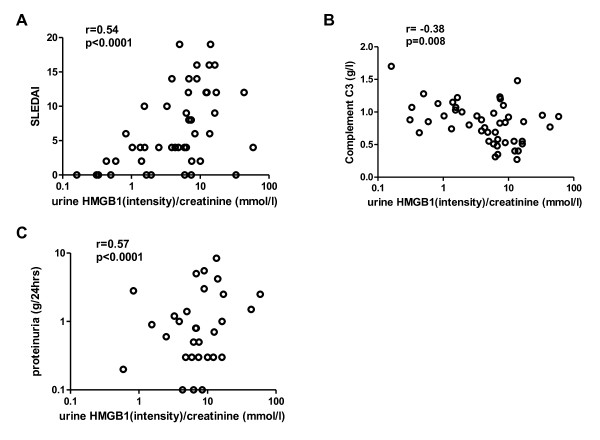
**Correlation of urinary high mobility group box 1 (HMGB1)/creatinine (Cr) ratios in systemic lupus erythematosus (SLE) patients with systemic lupus erythematosus disease activity index (SLEDAI), complement levels C3, and proteinuria**. HMGB1/Cr ratios correlate positively with SLEDAI scores (**A**), and proteinuria (**C**) and inversely with C3 (**B**).

**Figure 3:**
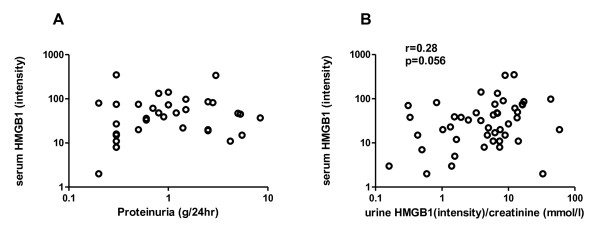
**Correlation of serum levels of high mobility group box 1 (HMGB1) with proteinuria and urinary HMGB1/creatinine (Cr) ratios**. (**A**) Serum HMGB1 levels are not significantly correlated to proteinuria. (**B**) Positive correlation between serum HMGB1 and urine levels of HMGB1.

Since urine levels of HMGB1 were increased in SLE patients, particularly in patients with active LN, we investigated whether urine levels of HMGB1 were related to clinical and serological parameters of disease activity, and might thus be used as an additional marker for assessment of disease activity, in particular renal activity. We observed a positive correlation between urine HMGB1/Cr ratios and SLEDAI (r = 0.54, *P *< 0.0001*) *(Figure 2A). Also, a significant negative correlation was observed between levels of C3 and urinary HMGB1/Cr ratios (r = -0.38, *P *= 0.008) (Figure 2B). A positive correlation was observed between urine HMGB1/Cr ratios and proteinuria (r = 0.57, *P < 0.0001*) (Figure 2C). Next we assessed the relation between serum HMGB1 and proteinuria in order to analyze whether urinary HMGB1 passively results from proteinuria. In this group of patients, serum HMGB1 levels were not correlated to proteinuria (Figure 3A). However, urinary HMGB1 and serum HMGB1 levels were positively correlated (r = 0.28, *P *= 0.056) (Figure 3B).

### Release of nuclear HMGB1 in the kidney

**Figure 4:**
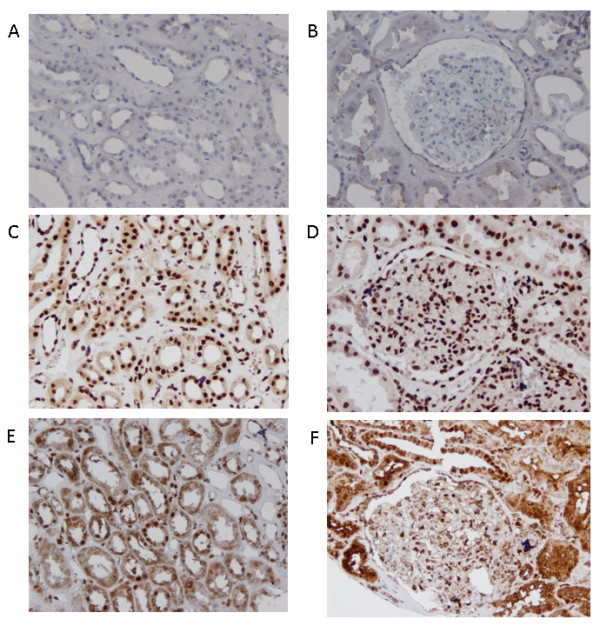
**Expression of high mobility group box 1 (HMGB1) in renal tissue of active lupus nephritis (LN) patients and controls**. Representative immunohistochemical staining of HMGB1 in a renal biopsy from an active LN patient (**E**,**F**) and control renal tissue (**C**,**D**). Isotype staining in control tissue (**A**,**B**). Renal tissue of the active LN patient shows a higher percentage of HMGB1-negative nuclei in tubular cells (**E**) and strong cytoplasmic and extracellular staining for HMGB1 in tubuli and glomeruli (**F**). Biopsy taken from normal renal tissue shows expression of HMGB1 mainly inside nuclei (**C**,**D**).

To assess the role of HMGB1 in renal inflammation or damage we assessed the presence and localization of HMGB1 in renal biopsies taken from patients with active LN in comparison to control renal tissue. In control renal tissue, HMGB1 was mainly present in the nuclei with minor cytoplasmic staining in the tubuli (Figure 4C) and in the glomeruli (Figure 4D). In biopsy sections from patients with LN, HMGB1 was no longer confined to the nuclei only, but was mainly expressed in the cytoplasm and the extracellular space (Figure 4E and 4F). In these patients, significant percentages of HMGB1 negative nuclei (resulting from HMGB1 release) were present. The percentage of HMGB1-negative nuclei was counted and data are summarized in Table 2. Although the glomerular structures of patients with active LN showed significant extracellular staining of HMGB1, only a few HMGB1-negative nuclei were detected in glomeruli. No correlation was found between the percentages of HMGB1 negative nuclei in renal tissue and levels of urinary HMGB1. We further assessed the association between the percentage of HMGB1 negative nuclei in the kidney, and histological findings such as activity index (AI) and chronicity index (CI) and renal function parameters (eGFR, creatinine and proteinuria). No correlation was found between histological scores and HMGB1 negative nuclei in the kidney (data not shown). Although no correlation was found between HMGB1 negative nuclei in the kidney and renal parameters (creatinine and proteinuria), there was a trend toward a negative correlation with estimated glomerular filtration rate (eGFR) (*r = -0.51, P *= 0.05).

### Expression of HMGB1 receptors (TLR2, TLR4 and RAGE) in the kidney of LN patients and controls

**Figure 5:**
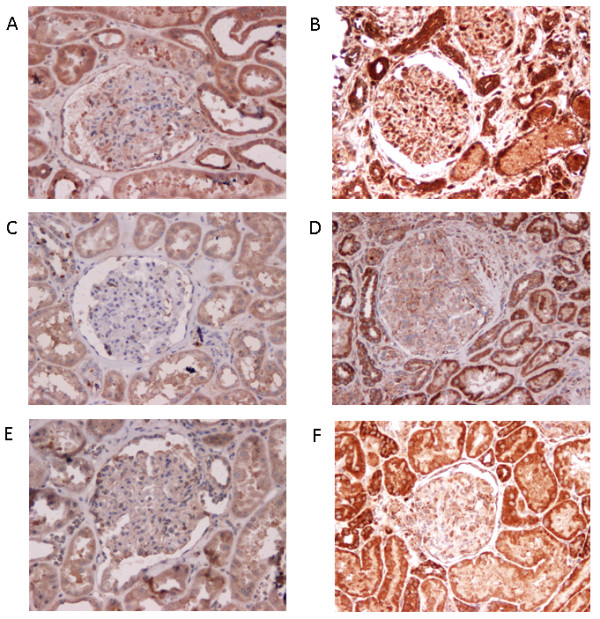
**Expression of high mobility group box 1 (HMGB1) receptors in a renal biopsy from a patient with active lupus nephritis (LN) and in control renal tissue**. Representative staining for RAGE, TLR2 and TLR4 in active LN (**D**,**E**,**F**) and control renal tissue (**A**,**B**,**C**), respectively. Renal tissue of an active LN patient shows strong cytoplasmic/extracellular expression for HMGB1 receptors in tubular cells, interstitial infiltrates, and glomeruli in comparison to control tissue.

As expression and release of HMGB1 was increased in the renal biopsies from LN patients, we further analyzed the expression of HMGB1 receptors, that is, TLR2, TLR4 and RAGE, in these biopsies as well as in control renal tissue. Control renal tissue showed relatively weak expression of TLR2, TLR4 and RAGE with 14% (12 to 16%) for TLR2, 14% (12 to 16%) for TLR4 and 0.2% (0.004 to 0.500%) for RAGE. In contrast, strong expression of TLR2, TLR4 and RAGE was observed in renal biopsies from patients with active LN compared to control renal tissue, with 28.7 (24.7 to 50.0%), *P *< 0.008, for TLR2; 28.8% (20 to 40%), *P *< 0.008, for TLR and 29% (17 to 40%), *P *< 0.008, for RAGE. Expression of TLR2, TLR4 and RAGE in active LN patients was mainly cytoplasmic and localized in glomeruli as wells as in tubular cells (Figure 5). Expression of various receptors was not associated with the percentage of HMGB1-negative nuclei in renal biopsies from patients with active LN, SLEDAI and renal function parameters.

## Discussion

In the present study we demonstrated for the first time that HMGB1 levels are significantly increased in the urine of SLE patients especially in those with active proliferative LN. Moreover, urinary levels of HMGB1 were correlated with disease activity scores, and reversely with levels of complement C3.

LN is a serious manifestation in patients with SLE that can progress to end-stage renal failure. Despite extensive studies, the pathogenesis of LN has not been fully clarified. Recently, the role of HMGB1 has been demonstrated in autoimmunity and chronic inflammatory diseases, especially those with renal manifestations such as antineutrophil cytoplasmic antibody (ANCA)-associated vasculitis, chronic kidney disease (CKD) and SLE [15,17,20-25]. However, until now no study has been conducted to evaluate urinary levels of HMGB1 as a reflection of renal inflammation. Thus, in the current study we investigated urine levels of HMGB1, in comparison to serum levels, in SLE patients with and without renal involvement. In accordance with our previous study, serum levels of HMGB1 were significantly increased in active LN patients compared to patients without active renal disease and controls [15]. Similarly, urinary levels of HMGB1 were increased in patients with active LN. Of note, urinary HMGB1 levels were also detectable, but at a lower level, in patients without active LN, but not in HC. Detectable amounts of HMGB1 in the urine of patients without active LN might be explained in two ways. A possibly ongoing low-grade renal inflammatory activity could contribute to the release of HMGB1. Indeed, Zickert *et al*. showed that extracellular expression of HMGB1 is detectable in renal biopsies taken from quiescent SLE patients at follow up [26]. Furthermore, some patients with SLE may have negative urine findings despite histological evidence of lupus nephritis [27]. Second, increased levels of serum HMGB1 might lead to urinary excretion of HMGB1, particularly in patients with a history of LN and slight persisting proteinuria. HMGB1 has been recognized as a new autoantigen and as an important inflammatory mediator in SLE as exemplified by increased serum levels and presence of antibodies against this protein [15-17]. Both HMGB1 and anti-HMGB1 antibodies have been shown to be associated with SLE disease activity, decreased complement levels, and proteinuria [15]. In our present study however, we did not find a positive correlation of HMGB1 serum levels with proteinuria. Now a large group of patients with newly diagnosed active LN without induction therapy was included in contrast to our previous study. This may explain the discrepancy in findings between both studies. Nevertheless, the absence of a correlation in the present study is not fully explained.

Interestingly, in the current study urinary HMGB1 levels were correlated with SLEDAI, particularly in patients with active LN. Furthermore, urinary levels of HMGB1 were inversely correlated with complement levels. We were not able to determine prospectively if urine levels of HMGB1 can be used for monitoring renal disease activity in patients with active LN. Nevertheless, increased urine levels of HMGB1 might indicate that HMGB1 is an important inflammatory mediator and that urinary HMGB1 might be an additional biomarker for assessment of renal disease activity in SLE. However, more studies are needed to ascertain that urinary levels of HMGB1 are a clinically useful marker.

The exact events leading to glomerular inflammation and damage are still not known. Anti-dsDNA or anti-nucleosome antibodies, present in immune complexes, have been suggested as important players. It has been shown that HMGB1 is an important component of these circulating DNA-containing immune complexes [6]. HMGB1 is responsible for immune-inflammatory activity and for binding of DNA-containing immune complexes, containing anti-dsDNA and/or anti-HMGB1 antibodies, to renal tissue, causing injury. However, the origin of HMGB1, whether it is produced outside the kidney or locally within the inflamed kidney, is still unresolved. In the current study, the percentage of HMGB1-negative nuclei, probably resulting from HMGB1 release, was higher in renal tissue of patients with active LN compared to control renal tissue, suggesting active release of HMGB1 in pro-inflammatory processes within the kidney. Our results are in line with the findings of Qing *et al*. [6]. In their study, specific data on the characteristics of their LN patients were not mentioned, and levels of HMGB1 in serum were not detectable. Also, urinary levels of HMGB1 were not measured. We were not able to definitely identify the cells releasing HMGB1. Release of HMGB1 could result from infiltrating cells as indicated by immunohistochemical staining, but could also result from either activation or cell death of constitutive renal tissue. Also, we cannot exclude the possibility that at least some of urinary HMGB1 might emerge from systemic inflammation. This might explain the lack of correlation between urinary HMGB1 and HMGB1 released from nuclei in the kidney. Further support for the role of HMGB1 in renal inflammation comes from the increased expression of the HMGB1 receptors TLR2, TLR4 and RAGE in the renal tissue of LN patients. To the best of our knowledge, this is the first study describing expression of the functional receptors (TLR2, TLR4 and RAGE) of HMGB1 in lupus nephritis. The low intrinsic expression of these receptors in control tissue suggests that TLR2, TLR4, and RAGE are all part of the innate immune system in the kidney. The intense and altered expression of TLR2, TLR4, and RAGE in patients with active LN, however, could reflect a role of these receptors in the development and severity of lupus nephritis. In combination, all these data indicate that HMGB1 might be an important player in the pathogenesis of LN and, possibly, a therapeutic target. Indeed, Mao *et al*. demonstrated that targeting HMGB1 by monoclonal antibodies in a murine adenovirus-accelerated SLE model inhibited the development of proteinuria and proved protective [28]. Clinical studies targeting HMGB1, however, have not been performed.

The present study provides evidence that HMGB1 expression is increased not only in the sera but also in the urine of active LN patients as well as at the site of local renal inflammation. Urine levels of HMGB1 could reflect both local and systemic inflammation. Urinary biomarkers might be more relevant than serum biomarkers because they may directly reflect renal pathology. However, further studies are needed to evaluate the clinical significance of measuring urinary HMGB1 and its value as a biomarker in lupus patients with renal involvement.

## Conclusions

The present study demonstrated increase in urine HMGB1 levels in SLE patients, in particular in those with active LN. Increase in urine HMGB1 levels correlated to SLE disease activity index (SLEDAI). Accordingly, renal tissue of LN patients showed increase in nuclear HMGB1 release compared to control tissue. Furthermore, expression of TLR2, TLR4 and RAGE was increased in a renal biopsy from active LN patients. Together, we suggest that HMGB1 may play an important role in renal pathology in SLE patients.

## Abbreviations

ACR: American College of Rheumatology; AI: activity index; ANCA: antineutrophil cytoplasmic antibody-associated vasculitis; anti-dsDNA: anti-double stranded DNA; C3: Complement 3; C4 Complement 4; CI: chronicity index; CKD: chronic kidney disease; Cr: creatinine; CRP: C-reactive protein; eGFR: estimated glomerular filtration rate; ELISA: enzyme-linked immunosorbant assay; HMGB1: high mobility group box 1; LN: lupus nephritis; LPS: lipopolysaccharide; RAGE: receptor for advanced glycation end products; SLE: systemic lupus erythematosus; SLEDAI: systemic lupus erythematosus disease activity index; TLRs: toll like receptors.

## Competing interests

The authors declare that they have no competing interests.

## Authors' contributions

DAA, JW and MB designed the study and DAA and JB did the HMGB1 measurements. SD collected patients' materials and MCvD performed biopsy preparation and classification. DAA, JW, JB, PCL, CGMK and MB were involved in interpretation of the results, while DAA, JW, MB and CGMK critically prepared the manuscript. All authors read and approved the final manuscript.
